# Iron Phosphide Nanobundles for Efficient Electrochemical Hydrogen Evolution Reaction in Acidic and Basic Media

**DOI:** 10.1021/acsami.4c09660

**Published:** 2024-10-29

**Authors:** Shubham Sharma, Nishan Khatri, Sharad Puri, Menuka Adhikari, Phadindra Wagle, David N. McIlroy, A. Kaan Kalkan, Yolanda Vasquez

**Affiliations:** † Department of Chemistry, Oklahoma State University, Stillwater, Oklahoma 74078, United States; ‡ Department of Mechanical and Aerospace Engineering, Oklahoma State University, Stillwater, Oklahoma 74078, United States; § Department of Physics, Oklahoma State University, Stillwater, Oklahoma 74078, United States

**Keywords:** iron phosphide, nanobundles, synthesis, electrochemical, hydrogen evolution reaction, water splitting, earth-abundant catalyst

## Abstract

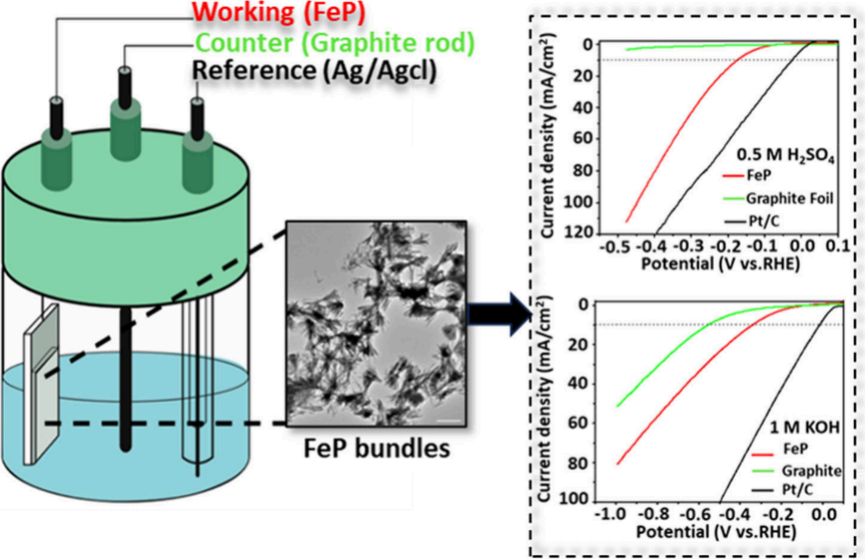

Earth-abundant transition metal phosphide (TMP) nanomaterials have gained significant attention as potential replacements for Pt-based electrocatalysts in green energy applications, such as the hydrogen evolution reaction (HER), oxygen evolution reaction (OER), and overall water splitting. In particular, FeP nanostructures exhibit superior electrical conductivity and high stability. Moreover, their diverse composition and unique crystal structures position FeP nanomaterials as emerging candidates for HER electrocatalysts. However, the synthesis or fabrication method employed for FeP nanostructures can significantly affect their overall electrocatalytic properties. For example, the solution synthesis of pure-phase FeP nanostructures remains challenging due to the formation of multiple binary phases and undesirable agglomeration. In this work, we use a simple approach to synthesizing FeP nanobundles by reacting β-FeOOH (iron oxyhydroxide) with trioctylphosphine (TOP). FeP nanobundles were evaluated as HER electrocatalysts in both acidic and basic conditions, demonstrating good HER activity with overpotential values of 170 and 338 mV at a current density of −10 mA cm^–2^ in acidic and alkaline solutions, respectively. Additionally, they exhibited low values of Tafel slopes in both acidic and alkaline environments. In acidic media with a pH of 0.45, the nanobundles showed no signs of deterioration for up to 15 h (−50 mA cm^−2^). In basic media with a pH of 13.69, the nanobundles remain stable for up to 8 h (−50 mA cm^−2^). These results demonstrate a simple and effective method for producing highly efficient earth-abundant and cost-effective TMP-based electrocatalysts, which could play a vital role in the hydrogen economy of the future.

## Introduction

Research on clean renewable fuels has been ongoing due to the rising demand for energy and the need to reduce carbon emissions.^1−3^ Hydrogen energy is considered an important and potentially consequential substitute to conventional fossil fuels due to its high energy density, which is about three times greater than gasoline.^4,5^ Molecular hydrogen can be produced through steam reforming, microbial fermentation, and photo- and electrocatalytic water splitting.^5−7^ The utilization of the cathodic half-reaction, known as the hydrogen evolution reaction (HER) in the electrolytic water splitting (2H_2_O → 2H_2_ + O_2_) process, is widely considered the most straightforward and sustainable method for producing high-purity molecular hydrogen on a large scale.^8,9^ However, the main obstacle for HER lies in the need for a high overpotential to generate a high current density due to the sluggish reaction kinetics and unfavorable thermodynamics.^8,10^ Currently, Pt, Pd/PtRu (111), Ir, and Ru based materials are considered the benchmarks for HER electrocatalysts, exhibiting lower overpotentials and wider pH adaptability;^11−13^ however, their limited abundance and high cost restrict their widespread use.^14,15^ Transition-metal sulfides, phosphides, nitrides, carbides, hydroxides, and bimetallic compounds are low-cost, earth-abundant alternatives that have gained considerable attention due to their favorable electrocatalytic performance.^1,16,17^ Among the diverse array of alternatives, transition-metal phosphides (TMPs) are considered the most promising inexpensive, earth-abundant electrocatalysts for HER due to their fast charge transfer kinetics, high electric conductivity, and other desirable properties.^17,18^ In TMPs, the negatively charged P atoms can capture positively charged protons, while the positively charged metal atoms serve as hydride-acceptor centers. Additionally, the unoccupied 3d orbitals or the lone pair of electrons in the 3p orbital of the phosphorus atoms can influence the charge of the iron atoms at the surface.^19,20^ These ensemble effects significantly enhance the performance of TMPs toward the HER.

The active centers of Fe in FeP are structurally and electronically similar to the active sites present in highly efficient biological HER enzymatic catalysts, specifically [FeFe]-hydrogenases. These centers facilitate an optimal hydrogen adsorption energy, resulting in faster kinetics.^10^ Although FeP is a good HER catalyst, the material’s morphology and scale (e.g., bulk, thin film, nanostructured) significantly affect its catalytic properties.^21^ One major drawback of the solution synthesis of nanoscale FeP materials is the formation of various phases (e.g., FeP_2_, Fe_3_P, etc.).^22,23^ Significant efforts have been devoted to the development of facile approaches for the synthesis of phase-pure FeP nanoparticles,^22,24−28^ most of which employed commercially available Fe-based organometallic precursors.

Recently, as an alternative to the above efforts, we reported the synthesis of iron phosphide (FeP) nanobundles through solution-based thermal decomposition of iron oxyhydroxide (β-FeOOH) in trioctylphosphine (TOP).^29^ This study shows that the heating rate influences the transformation of β-FeOOH into FeP. Slower rates (4.5 °C/min) result in incomplete transformation, suggesting a kinetic barrier, possibly due to the formation of Fe_2_P. Interestingly, a fast heating rate (18.8 °C/min) shifts the equilibrium to favor FeP as the major product. In this report, we evaluate the electrocatalytic activity of FeP nanobundles toward the hydrogen evolution reaction (HER). The FeP nanobundles demonstrate notable HER performance, with low overpotential values of 170 and 338 mV at a current density of *j* = −10 mA/cm^2^. Additionally, Tafel slopes of 75 and 159 mV/decade were measured in 0.5 M H_2_SO_4_ and 1 M KOH, respectively. The HER performance of FeP nanobundles positions them as a promising and cost-effective electrocatalyst for hydrogen economy.

## Experimental Section

### Materials

Iron(III) chloride hexahydrate (FeCl_3_·6H_2_O, ≥98% ACS grade), a 50% (w/v) poly(ethylenimine) solution (PEI, MW = 750000), and trioctylphosphine (TOP, P(C_8_H_17_)_3_, ≥90% technical grade) were purchased from Sigma-Aldrich (St. Louis, MO). Anhydrous ethyl alcohol 200 proof (absolute, ACS/USP grade) and hexanes (ACS/USP grade) were purchased from Pharmco (Brookfield, CT). Transmission electron microscopy (TEM) Cu grids (carbon-coated, 200 mesh) were purchased from Electron Microscopy Sciences (Hatfield, PA).

### Preparation of Iron Oxyhydroxide (β-FeOOH) Nanoneedles

Iron oxyhydroxide nanoneedles were prepared using a simple hydrolysis method previously reported in the literature with some minor modifications.^30−32^ Typically, 5.4 g (20.0 mmol) of solid FeCl_3_·6H_2_O was dissolved in 100 mL of DI water (18.2 MΩ·cm) at room temperature in a 500 mL three-necked round-bottom flask fitted with a condenser. Next, 620.7 μL of a 47.5% v/v PEI solution were added dropwise to the reaction mixture while stirring (400 rpm). The reaction was maintained at 80 °C in an oil bath for 2 h. The brownish-yellow precipitate was collected by high-speed centrifugation at 8000 rpm for 15 min, washed several times with ethanol, and dried overnight in a vacuum desiccator (Nalgene). The dimensions of the β-FeOOH nanoneedles were approximately *l* = 90 ± 15 nm and *w* = 12 ± 4 nm, as measured from the TEM images. ImageJ (1.5d) software was used to process TEM images.

### Preparation of FeP Nanobundles

The synthesis of FeP nanostructures involves two steps: (i) the synthesis of iron oxyhydroxide (β-FeOOH) nanoneedles and (ii) the subsequent conversion of β-FeOOH nanoneedles to FeP on treatment with TOP. A reaction mixture of 0.059 g of β-FeOOH and 3.96 mmol of TOP was heated at a rate of 18.8 °C/min to reach 320 °C within 17 min from room temperature. Afterward, the reaction mixture was maintained at 320 °C for 4.5 h under an argon atmosphere with continuous stirring (600 rpm). After cooling the system to room temperature, the final product was isolated by adding excess ethanol (10–20 mL) and centrifuging at 8000 rpm for 2 min to isolate the solid particles. The black solid was washed 6 times with hexanes and chloroform until the supernatant was clear. The FeP particles were dried in a vacuum desiccator (Nalgene) overnight.

### Characterization Techniques

The morphology and size of the resulting nanobundles were determined with a JEOL JEM 2100 transmission electron microscope (TEM) at an accelerating voltage of 200 kV and a beam current of 102 μA. Powder X-ray diffraction (pXRD) patterns of the product were acquired with a Rigaku Smart Lab X-ray diffractometer with a Cu Kα radiation source (λ = 1.54 Å). The 2θ scan range was varied from 5° to 90° at a scan rate of 5°/min. X-ray photoelectron spectroscopy (XPS) was conducted with a base pressure of less than 10^–10^ Torr at ambient temperature. The spectra were obtained using the Al Kα emission line from a dual-anode X-ray source (PREVAC XR 40B) operated at 405 W with an angle of incidence of 54.7° and normal emission. The kinetic energy of the photoelectrons was acquired with an Omicrometer EA 125 hemispherical electron energy analyzer with a resolution of 0.025 eV. The textural properties of the resultant nanobundles were evaluated by N_2_-sorption analysis using Quantachrome AUTOSORB-1 (AS1-11). Raman spectroscopy was performed using a WITec alpha 300R Raman microscope, employing 532 nm laser excitation, 600 lines/mm grating, and a 100 μm confocal aperture (fiber) diameter. The Raman spectrum of the as-synthesized nanobundles was acquired using a 100× objective lens of 0.9 numerical aperture. The signal was integrated for 400 s. The laser power and beam spot size on the sample were set to 0.5 mW and 1 μm, respectively. FTIR spectra were acquired by using a Thermo Scientific Nicolet spectrometer. Inductively coupled plasma optical emission spectroscopy (ICP-OES) was conducted on the collected electrolyte samples in the Soil, Water, and Forage Analytical Laboratory. Scanning electron microscopy (SEM) images were taken on an FEI Quanta 600 field emission gun ESEM with Bruker energy-dispersive X-ray spectroscopy capabilities.

### Electrochemical Experiments

The electrochemical measurements were performed with a Gamry potentiostat (Interface 1000-11122A) electrochemical workstation using a standard three-electrode configuration consisting of a graphite-foil-based working electrode (1 × 1 cm^2^), a graphite rod counter electrode, and a double junction silver/silver chloride (Ag/AgCl) reference electrode. To prepare the graphite-foil-based working electrode, a slurry was prepared by combining FeP nanobundles as the active material (70 wt %, 23.3 mg), conducting carbon (Super P, Alfa Aesar, USA, 15 wt %, 5.0 mg), poly(vinylidene fluoride) as a binder (Thermo Scientific, USA, 15 wt %, 5.0 mg), and *N*-methylpyrrolidone as solvent (NMP, Acros Organic, USA, 40 μL). The slurry mixture was stirred magnetically for 24 h at room temperature (25 °C) to improve the uniformity. A working electrode with a mass loading of 0.85 mg/cm^2^ was prepared by drop-casting the slurry on the graphite foil and drying overnight at 70 °C. The backside of the working electrode was insulated with electrical tape in all measurements. All the potentials in this work were reported against the reversible hydrogen electrode (RHE) using the relationship *E*_RHE_ = *E*_Ag/AgCl_ + 0.197 V + 0.059pH. The HER performance of the synthesized FeP nanobundle electrodes was evaluated by linear sweep voltammetry (LSV), electrochemical impedance spectroscopy (EIS), and chronopotentiometry. The double-layer capacitance was measured by using cyclic voltammetry (CV).

## Results and Discussion

**Figure 1:**
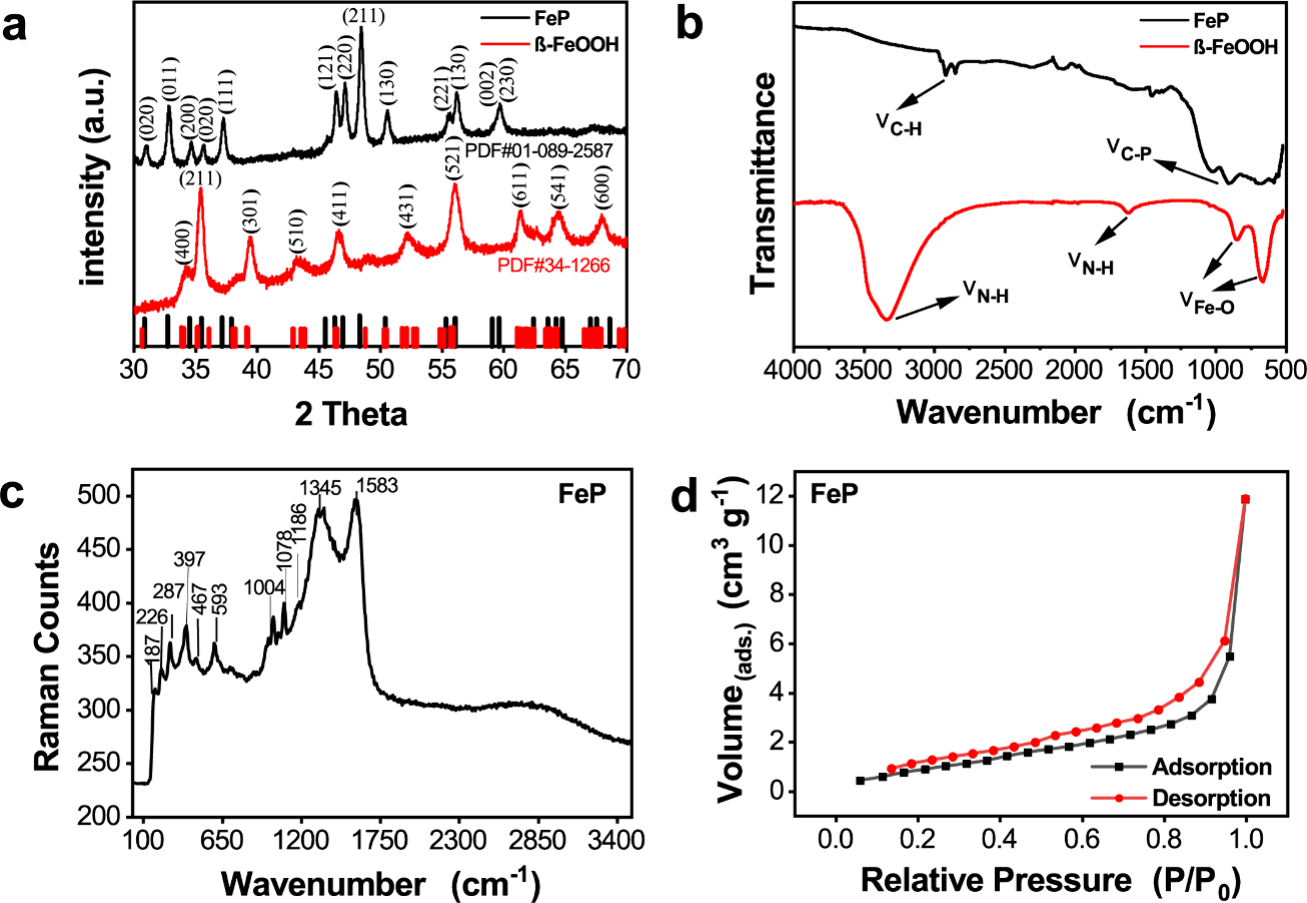
Characterization of FeP nanobundles synthesized from β-FeOOH nanoneedles and trioctylphosphine: (a) pXRD patterns of β-FeOOH (red pattern, JCPD 34-1266) and FeP (black pattern, JCPDS No. 01-089-2587); (b) FTIR spectra of β-FeOOH (red pattern) and FeP (black pattern); (c) Raman spectrum of FeP; (d) N_2_ sorption isotherm measured at 77 K for FeP.

FeP nanobundles were synthesized by reacting iron oxyhydroxide (β-FeOOH) nanoneedles with TOP as a phosphorus source at elevated temperatures and were characterized by various techniques. The pXRD patterns in Figure 1a reveal a phase transformation from monoclinic β-FeOOH to orthorhombic FeP. The reflections indexed to the (020), (011), (200), (020), (111), (121), (220), (211), (130), (221), (130), (002), and (230) atomic planes are indicative of the orthorhombic phase of FeP (JCPDS No. 01-089-2587).^33^ The purity of FeP is further evaluated by structural refinement via the Rietveld method. The crystallographic parameters derived from the Rietveld refinement are listed in Table S1. Figure S2 depicts the crystallographic arrangement of FeP nanobundles, confirming their adherence to the orthorhombic structure within the *Pbnm* space group (62: *Pbnm*). In this structure, each Fe^3+^ ion is coordinated to six equivalent P^3–^ ions, forming a network of distorted edge-, face-, and corner-sharing FeP_6_ octahedra, and these distorted P^3–^ actively form moderate bonds with reaction intermediates, creating proton- and hydride-acceptor centers that enhance the HER.^34,35^Figure 1b shows the FTIR spectra of the FeP nanobundles and β-FeOOH nanoneedles. The FTIR spectrum for β-FeOOH nanoneedles shows a distinct absorption band at ∼3400–3500 and 1500–1700 cm^–1^ corresponding to −N–H stretching from the PEI surface stabilizer, whereas the absorption band at ∼800–1000 cm^–1^ corresponds to −Fe–O stretching and is consistent with iron oxyhydroxide. The FTIR spectra of FeP nanobundles exhibit absorption bands at ∼2850 and ∼2960 cm^–1^ that correspond to C—H stretching modes from the alkylphosphine, while the absorption bands at ∼1036 and ∼1100 cm^–1^ are attributed to the C—P stretching bands of trioctylphosphine (TOP). In-situ catalytic cleavage of P—C bonds of the TOP molecules generates the active phosphorus species that reacts with β-FeOOH nanoneedles to generate FeP.^36,37^

Figure 1c shows a representative Raman spectrum of the as-synthesized FeP nanobundles. The peaks at 187 and 397 cm^–1^ are assigned to the Raman-active B_2g_ phonon modes, and the peaks at 226 and 287 cm^–1^ are assigned to A_g_ modes in FeP.^38^ The nanobundles consist of a minor fraction of FeP_2_ domains, as inferred from the weak peak at 467 cm^–1^ assigned to the B_1g_ mode in FeP_2_.^39^ The presence of FeP_2_ cannot be attributed to a phase transformation under Raman laser irradiation (e.g., due to the photothermal effect) because the 467 cm^–1^ peak disappears when the laser intensity is tripled. The FeP nanobundles exhibit the characteristics of weak Raman scattering with strong optical absorption. As a result, photothermal oxidation occurs even at low laser power (e.g., 0.5 mW) at the threshold of FeP detection, albeit slowly. We observe the emergence and systematic evolution of phosphate and phosphite phases in nanobundles as photoproducts. The peaks at 593 and 1004 cm^–1^ are assigned to the bending of a PO_4_ network and symmetric PO_4_-stretching modes in α-FePO_4_, respectively.^40,41^ Moreover, the peaks at 1078 and 1186 cm^–1^ arise from the PO_3_ and PO_2_-stretching modes in Fe_7_(PO_4_)_6_ and FePO_3_, respectively. While oxidation of FeP may also occur in ambient conditions, the detected phosphate and phosphite Raman peaks can only be attributable to the Raman laser exposure.^42,43^ The Raman spectrum shown in Figure 1c also suggests the presence of highly disordered and soot-like graphitic (sp^2^) carbon particles as identified from the D-band (1345 cm^–1^) and G-band (1583 cm^–1^).^44,45^ The graphitic carbon growth suggests in-situ thermolysis of TOP. The D and G bands of graphitic carbon persist even after washing 6 times with chloroform. The dominance of the carbon signal in the Raman spectrum is due to the high resonant Raman cross section of sp^2^ carbons, whereas FeP has a much lower Raman cross section due to its lower polarizability and high density of free electrons. The electron–electron collisions outcompete the resonant Raman process in FeP.

N_2_ isotherm measurements were conducted at 77 K to evaluate the specific surface area and porosity of the FeP nanobundles, as shown in Figure 1d. The FeP nanobundles show a Type I N_2_ isotherm, consistent with a microporous structure.^46^ The narrow hysteresis in the high-pressure region between a *P*/*P*_0_ ratio of 0.40 and 0.85 indicated a hierarchical pore structure.^47^ The BET surface area was 4.12 m^2^ g^–1^ with a total pore volume of 0.018 cm^3^ g^–1^ at a *P*/*P*_0_ ratio of 0.99 (Table S2). The presence of hysteresis in the nitrogen isotherms down to a low *P*/*P*_0_ ratio of 0.20 suggests the presence of narrow slit pores or bottle-shaped pores within the FeP nanobundles.^48^ These hierarchical pores facilitate access to catalytic sites and improve phase boundary contact and gas release during the hydrogen evolution reaction (HER), thereby enhancing overall HER activity.^49^

**Figure 2:**
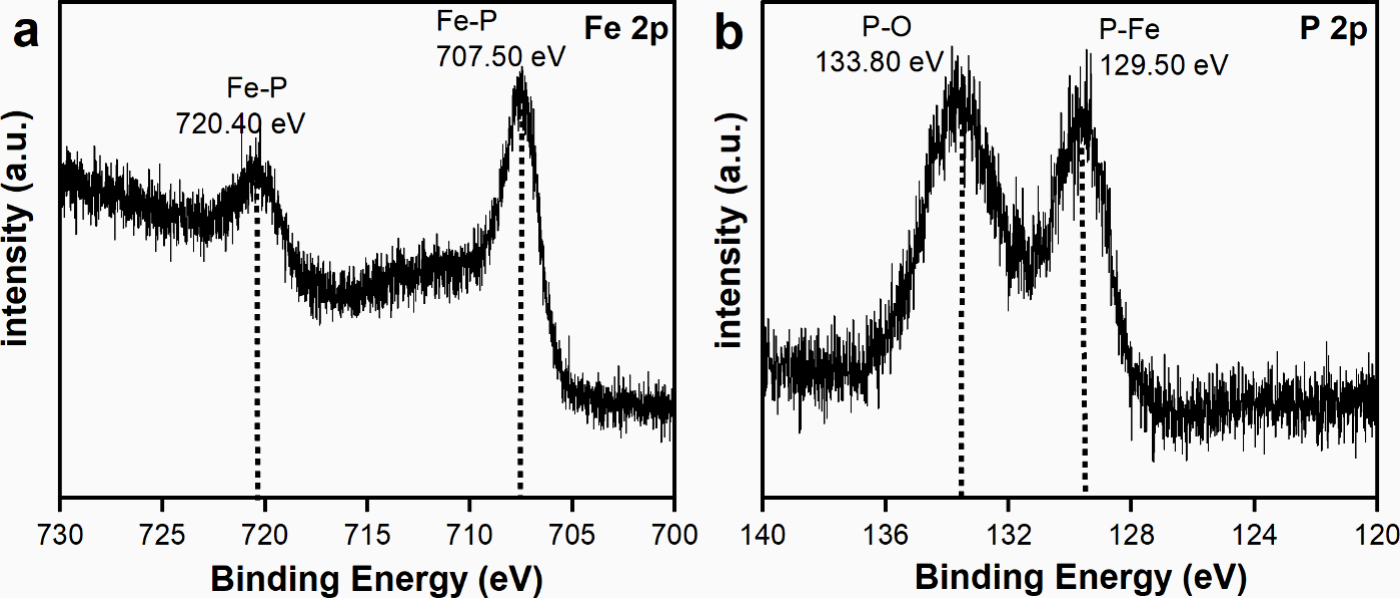
High-resolution XPS core level spectra of the (a) Fe 2p and (b) P 2p regions of the as-synthesized FeP nanobundles.

FeP samples were further evaluated by X-ray photoelectron spectroscopy (XPS) to verify chemical and surface composition. (The survey spectra are shown in Figure S3.) Figure 2 shows the high-resolution XPS core level spectra of the Fe 2p and P 2p regions. The Fe 2p_3/2_ and Fe 2p_1/2_ peaks centered at 707.5 and 720.4 eV, respectively, are consistent with previous reports of FeP.^50,51^ The high-resolution P 2p region exhibited two peaks centered at 129.5 and 133.8 eV, which correspond to the P^3–^ anion (2p_3/2_) in FeP and an oxidized phosphorus species that results from surface oxidation of FeP.^52,53^ The Fe 2p_3/2_ signal centered at 707.5 showed a 0.7 eV increase in binding energy compared to the neutral Fe metal peak, and the P 2p_3/2_ peak at 129.5 eV exhibited a 0.7 eV decrease in binding energy compared to elemental phosphorus (130.2 eV), indicating a transfer of electron density from Fe to P.^54,55^ The positive iron and the negative phosphorus sites facilitate the adsorption of reactants and the release of products, acting as sites that accept hydrides and protons, respectively, during the HER. Hence, this ensemble effect provides a framework that has the potential to enhance the catalytic process of the HER.^24,51^

**Figure 3:**
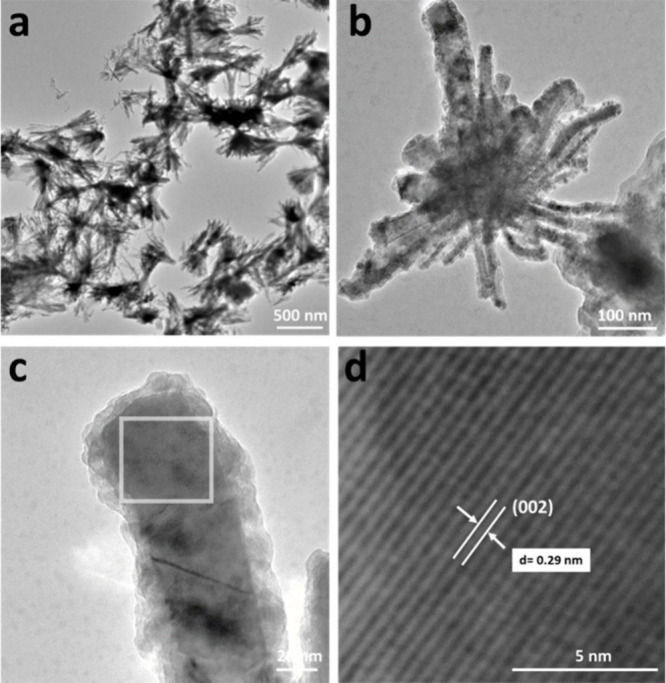
TEM images of FeP. (a) Low-magnification image of FeP nanobundles. (b) High-magnification image of FeP reveals that the nanobundles are polycrystalline. (c) An image of an individual branch of FeP. (d) Lattice fringes indicate a lattice spacing of *d*_002_ = 0.29 nm consistent with the orthorhombic phase of FeP.

The FeP nanobundles were characterized by using transmission electron microscopy (TEM), as presented in Figure 3. The images showed that the particles were polycrystalline and exhibited bundled or quasi-bundled morphologies (Figures 3a and 3b). The selective area electron diffraction (SAED) diffraction rings correspond to the (132), (600), and (234) planes of the orthorhombic phase of FeP (Figure S4). High-resolution transmission electron microscopy (HRTEM) analysis of a single branch of a bundle (Figure 3c) revealed a lattice spacing of 0.29 nm consistent with the (002) plane of the orthorhombic phase of FeP.^18,55,56^ Additionally, a lattice spacing of 0.26 nm, consistent with the (020) planes, is shown in Figure S5.

**Figure 4:**
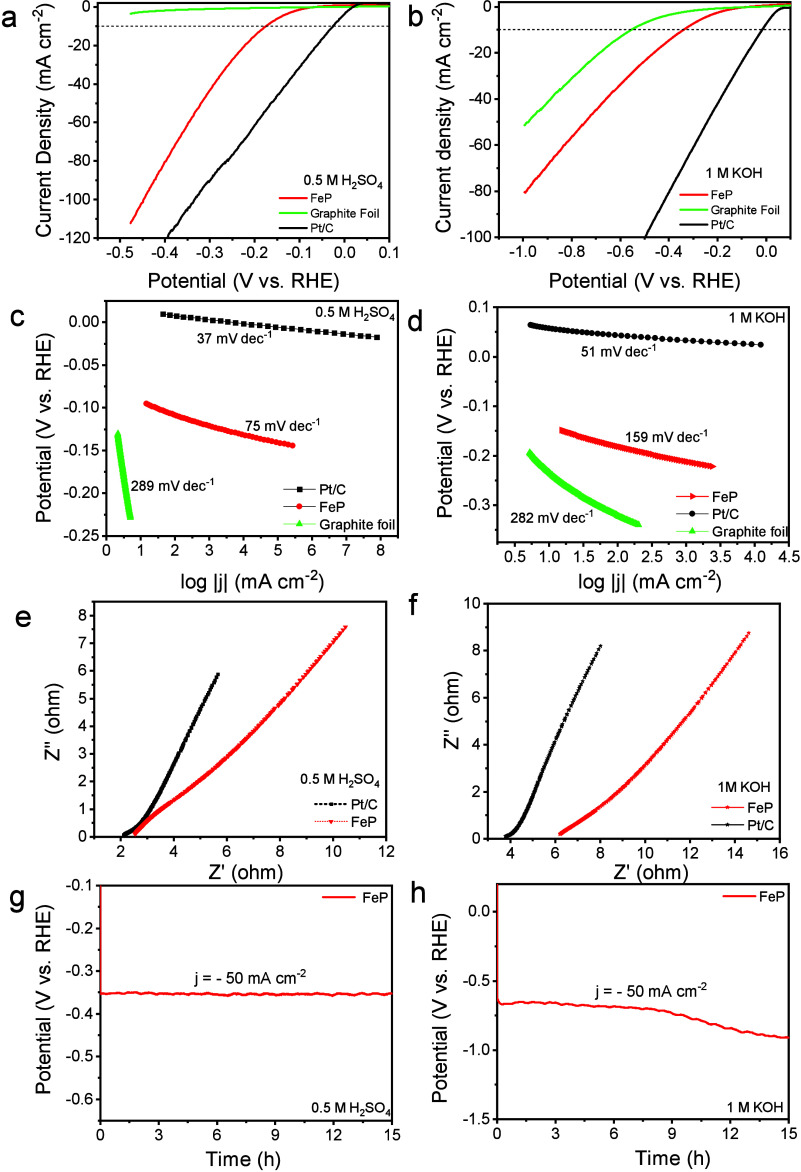
HER performance of FeP nanobundles. Linear sweep voltammetry (LSV) curves of the FeP nanobundles of bare graphite and Pt/C in (a) a 0.50 M H_2_SO_4_ and (b) a 1 M KOH solution. Tafel plots of FeP nanobundles, bare graphite, and Pt/C in (c) 0.50 M H_2_SO_4_ and (d) 1 M KOH solutions. Nyquist plots of FeP and Pt/C electrodes evaluated from 10 kHz to 1 Hz in (e) 0.50 M H_2_SO_4_ and (f) 1 M KOH solutions. Chronopotentiometric curves for FeP at constant current density (*j* = – 50 mA/cm^2^) for 15 h in (g) 0.50 M H_2_SO_4_ and (h) 1 M KOH solutions.

We then examined the electrochemical performance of FeP nanobundles coated on graphite foil electrodes in 0.5 M H_2_SO_4_ and 1 M KOH solutions. The catalytic activity of bare graphite foil and commercially available Pt/C (20 wt %, Thermo Scientific) was also evaluated for comparison. Figures 4a and 4b represent the linear sweep voltammetry curves (*j*–*V* plot) of FeP (mass loading = 0.85 mg/cm^2^), bare graphite foil, and Pt/C (20 wt %) in 0.5 M H_2_SO_4_ and 1 M KOH solutions, respectively. As anticipated, Pt/C (20 wt %) demonstrated an effective HER activity, displaying a low onset overpotential. In contrast, the unmodified graphite foil electrode exhibited poor catalytic performance, demanding overpotentials of beyond 500 mV for achieving a current density of *j* = −10 mA cm^–2^ in 0.5 M H_2_SO_4_ and 1 M KOH. The FeP-coated graphite foil shows an overpotential value of 170 and 338 mV in 0.5 M H_2_SO_4_ and 1 M KOH, respectively, to afford a current density of *j* = −10 mA cm^–2^. Beyond the measured overpotentials, the cathodic current density increased rapidly toward more negative potentials. The HER performance of FeP nanobundles is found to be comparable to those of previously reported TMP and non-noble-metal catalysts.^18,51,57,58^Tables S3 and S4 show a comparison of the overpotential and Tafel slope values of various TMP and non-noble-metal electrocatalysts. The catalytic activity of FeP nanobundles could be enhanced by their unique morphology. First, high surface-to-volume increases the exposed catalytic active sites for hydrogen adsorption on the catalyst surface. Second, the high curvature of the nanobundles increases the electron injection efficiency due to enhanced local field (lightning rod effect).^59,60^ The lower value of charge-transfer impedance favors facile electrode kinetics.^61^ The HER polarization kinetics were measured and analyzed by the Tafel equation for FeP, bare graphite foil, and Pt/C (20 wt %) catalysts. Based on previous reports in the literature, HER in acidic and basic media follows the three-step mechanism as follows:^62^

(a) Volmer step:
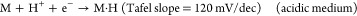

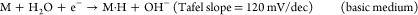
(b) Heyrovsky step:
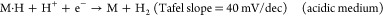

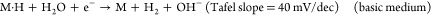
(c) Tafel step:
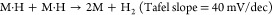
where M and M·H denote the active site of the catalyst surface without and with hydrogen adsorbate, respectively. Figures 4c and 4d show the Tafel plots (overpotential (η) vs log(*j*)) of FeP, bare graphite foil, and the Pt/C (20 wt %) catalyst in 0.5 M H_2_SO_4_ and 1 M KOH electrolytes, respectively. The Tafel slope (*A*) and exchange current density (*j*_0_) were calculated by fitting the linear region of the plot to the Tafel equation:
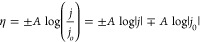
where η denotes the applied overpotential, *j* is the current density, *A* is the Tafel slope, and the equation intercepts the  axis at ).^55,63^ The positive and negative signs in the first terms of the above equation are for anodic and cathodic processes (oxidation and reduction), respectively. It is vice versa for the last term. Note that we have adapted the convention of negative current for reduction; therefore, the logarithm of the current density is expressed as . The Tafel slope, *A*, is defined as always positive in this study, which is why ± and ∓ signs are needed in the equation (i.e., the slope of the equation is negative for reduction). The Pt/C (20 wt %) shows a lower Tafel slope of 37 and 51 mV/decade in acidic and basic media, respectively, close to the value reported in the literature.^64,65^ The bare graphite electrode shows Tafel slopes of 289 and 282 mV/decade in 0.5 M H_2_SO_4_ and 1 M KOH, respectively. On the other hand, FeP nanobundles exhibit a Tafel slope of 75 and 159 mV/decade, respectively. The lower Tafel slope value of FeP could result from the lower interfacial resistance, *R*_ct,_ value of the FeP-coated electrode. In addition, the nanobundle-like framework, with its higher curvature effect, likely exposes more Fe and P active sites, exhibiting an ensemble effect wherein both hydride acceptors and proton acceptors are present to enhance the HER activity.^66,67^

**Figure 5:**
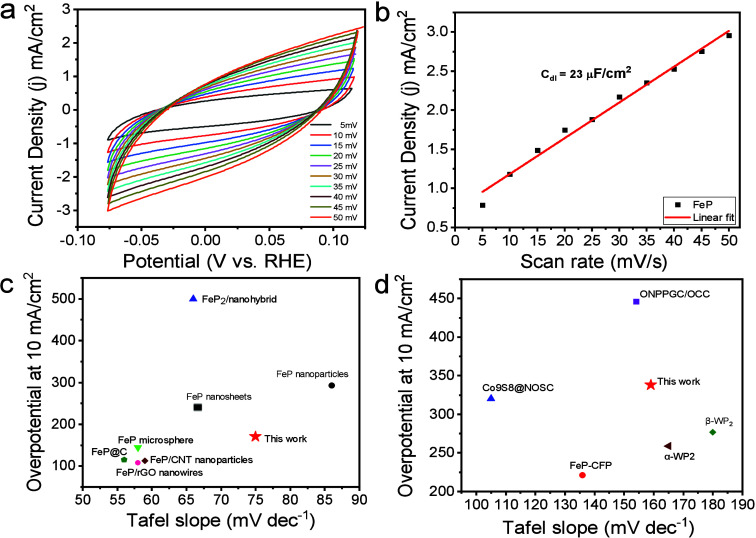
(a) CV curves of FeP nanobundles at various scan rates in the non-Faradaic region. (b) Correlation between current density and scan rate for determining the double-layer capacitance (*C*_dl_) of FeP nanobundle electrodes. Electrocatalytic HER performance comparison of FeP nanobundles with other iron-based phosphides and non-noble-metal electrocatalysts in (c) 0.5 M H_2_SO_4_ and (d) 1 M KOH.

Electrochemical impedance spectra (EIS) for FeP were also measured and compared with 20 wt % Pt/C, as shown in Figures 4e and 4f. The Nyquist plot shows no minuscule semicircle for the FeP electrocatalyst in 0.5 M H_2_SO_4_ and 1 M KOH electrolytes, indicating the low interfacial resistance (*R*_ct_) between the electrode and the electrolyte. The low interfacial resistance can be attributed to integrating a conductive electronic framework and the specific surface area exhibited by the FeP nanobundles. This combination provides an optimal environment for the effective adsorption of active (H*) species and the efficient desorption of (H_2_) moieties. Moreover, the abundance of active sites within the nanobundles facilitates the percolation of electrolytes into the electrode structure.^68^ The superior HER activity of the FeP nanobundles is attributed to the low-energy electron transfer kinetics at the electrolyte interface. In the case of 20 wt % Pt/C, no semicircle was detected in either acidic or basic media. This implies easy movement of electrons between the junction of the electrode and the solution, as described in the literature reports.^68,69^ The stability of the FeP electrodes was evaluated in acidic and basic media by using galvanostatic measurements (mass loading = 0.85 mg/cm^2^) at *j* = −50 mA/cm^2^. As shown in Figures 4g and 4h, the FeP nanobundle catalyst has long-term durability with negligible activity loss at a constant current density of *j* = −50 mA/cm^2^ up to 15 and 8 h in 0.5 M H_2_SO_4_ and 1 M KOH, respectively. A minor increase in the overpotential could be caused by the desorption of some FeP particles from the substrate, leading to a slight decrease in mass loading.^70^ To further elucidate the correlation between the number of active sites and electrocatalytic HER activity of FeP nanobundles, we measured the double-layer capacitance (*C*_dl_) using cyclic voltammetry (CV) curves at various scan rates (5–50 mV s^–1^) in the non-Faradaic region as shown in Figures 5a and 5b. The experimentally determined *C*_dl_ value of 23 μF/cm^2^ suggests that the unique nanobundle morphology possesses a substantial electrochemically active surface area (ECSA), which enhances the HER activity. Moreover, the electrocatalytic HER performance of our FeP nanobundles compares favorably with various morphologies of iron-based phosphides and other non-noble-metal electrocatalysts in both acidic and basic media (Figures 5c and 5d; full details in Tables S3 and S4).

### Post-HER Characterization of the FeP Nanobundle Electrodes and Electrolytes

We conducted Raman spectroscopy, XPS, and FE-SEM on the FeP electrodes of Figures S4g and S4h, as well as ICP-OES on their electrolytes, after the chronopotentiometry (−50 mA/cm^2^ for 15 h) to gain insight into the surface chemistry changes and leaching, if any. Because of the cathodic bias on the electrodes, no oxidation of FeP is expected, but reduction is possible. Both electrodes exhibit the strong D, G, and 2D Raman peaks of the activated carbon, which is 15 wt % (Figure S6). Interestingly, no Raman peaks are detectable from the poly(vinylidene fluoride) binder (15 wt %). Both electrodes show the characteristic FeP peaks confirming FeP is the major electrocatalyst during the chronopotentiometry. Reductive formation of a new phase or compound is not evidenced, but such accumulations could be below the detection limit. As observed during the Raman acquisition for powder FeP (Figure 1c), a distinct phosphate peak also emerges and systematically evolves during the Raman acquisition for the electrode immersed in H_2_SO_4_ (1012 cm^–1^), attributable to photooxidation due to the Raman laser. However, phosphate formation during HER, if any, is below the detection limit. Surprisingly, laser-induced evolution of the phosphate peak is absent for the electrode immersed in KOH (even if the laser power is increased to a maximum of 44 mW). Given that the HER overpotential increases beyond 8 h in KOH, we hypothesize the formation of a conformal layer. While such a layer may limit HER after 8 h, it may also be protecting FeP against oxidation during laser exposure. The ICP-OES analysis revealed 12.7 and 23.4 ppm P and 8.2 and 0.07 ppm Fe in the 0.5 M H_2_SO_4_ and 1 M KOH electrolytes after 15 h of chronopotentiometry, respectively (Table S5). The percentages of Fe and P leached from the electrode to the electrolyte were computed and are reported in the note of Table S5. Based on these findings, leaching of FeP is inferred to be not significant.

High-resolution XPS core level spectra (Figure S7) show the presence of P 2p peaks associated with FeP and PO_4_^3–^ for the pristine electrode, indicative of surface oxidation under ambient conditions (a survey XPS spectrum is also provided in Figure S8). Both peaks are attenuated after chronopotentiometry in 0.5 M H_2_SO_4_ and 1 M KOH, indicative of leaching in the thickness range of XPS, which is about 10 nm; however, the intensity ratio of FeP to PO_4_^3–^ remained the same. The intensity of the Fe 2p_3/2_ and Fe 2p_1/2_ peaks are reduced at a similar percentage as the P 2p peaks in 0.5 M H_2_SO_4_ after chronopotentiometry. However, in KOH, essentially no attenuation of the Fe peaks is observed, which we explain by the passivation of Fe by OH^–^. Similar behavior was observed for CoP_2_ electrodes in KOH where leaching rate of Co was significantly less compared with that of P during HER.^71^

FE-SEM was conducted to gain insights into the surface morphology of the FeP electrodes before and after chronopotentiometry (Figure S9). The electrodes maintain a similar microstructured topography after chronopotentiometry. The stoichiometry is not quite 1:1 Fe:P since the data are taken from agglomerates, not individual particles, and because of the presence of surface phosphates in the pristine sample (before chronopotentiometry) (Figure S10). Moreover, EDS data show the presence of Fe and P in both electrodes after chronopotentiometry in H_2_SO_4_ (Figure S11) and 1 M KOH (Figure S12). Elemental data show the presence of Al, Si, and Ca, which is attributed to contaminants from the etching of borosilicate glass partially during the synthesis of FeP (TOP is known to etch glass) but more so during the potentiometric experiment conducted in H_2_SO_4_. The presence of S is attributed to sulfate from H_2_SO_4_. As expected, high levels of K are seen in the EDS data for the electrode in KOH. There is also a lower atomic % P, which may be due to more phosphorus leaching from the electrode surface in the KOH electrolyte relative to H_2_SO_4_, consistent with ICP-OES data.

## Conclusion

FeP nanobundles show notable performance toward HER electrocatalysis with overpotentials of 170 and 338 mV at low mass loading and *j* = −10 mA/cm^2^ (appropriate operational current density) in 0.5 M H_2_SO_4_ and 1 M KOH solutions, respectively. Furthermore, FeP nanobundles are durable, exhibiting no signs of substantial degradation of electrocatalytic HER activity in the evaluated media at −50 mA/cm^2^ for 15 h. Based on our Raman spectroscopy, XPS, and ICP-OES characterizations, surface chemistry changes of the FeP electrodes are different in H_2_SO_4_ and KOH electrolytes during HER. Particularly, leaching of Fe is selectively impeded over that of P in KOH, which we owe to OH^–^ passivation. Additionally, the high surface-to-volume ratio of the FeP nanobundles provides an optimal energy pathway for electron transport and increases the number of catalytic sites per unit area of the electrode (projected area normal to the electrode surface). This catalyst shows an enhanced exchange current density, lower overpotentials, and notable catalytic activity toward HER. In addition, the outcomes of this study affirm that transition-metal phosphide electrocatalysts, being low cost, are promising and noteworthy contenders for the emerging hydrogen economy. The results suggest that TMP electrocatalysts could be an effective alternative to noble-metal catalysts for hydrogen evolution reactions. In future work, introducing metal doping and additional nanostructure morphologies could help enhance charge transport, and thus, the electrocatalytic activity of FeP nanobundles can be further improved toward HER.

## Supporting Information Available

Refined structure parameters and crystal structure for as-synthesized FeP nanobundles; XPS survey spectra; BET surface area analysis profile; a SAED pattern; a comparison table of previously reported iron phosphide and transition-metal phosphides (TMPs) in 0.5 M H_2_SO_4_ and 1 M KOH; post-HER Raman and XPS spectra; post-HER ICP-OES analysis of the electrolytes; FE-SEM images and energy dispersive X-ray spectra before and after HER (PDF)

## Abbreviations

TOP: trioctylphosphine
LSV: linear sweep voltammetry
EIS: electrochemical impedance spectroscopy
HER: hydrogen evolution reaction.
