# Supplementary material for: Iron Phosphide Nanobundles for Efficient Electrochemical Hydrogen Evolution Reaction in Acidic and Basic Media
Source: ACS Appl Mater Interfaces. 2024 Oct 29;16(45):61858–67. doi: 10.1021/acsami.4c09660 (PMC11565577; doi:10.1021/acsami.4c09660)
Supplement: Supplementary file 1 — am4c09660_si_001.pdf [file am4c09660_si_001.pdf]

Supporting Information

Iron Phosphide Nanobundles for Efficient Electrochemical Hydrogen Evolution Reaction in Acidic and Basic Media

Shubham Sharma,[1] Nishan Khatri,[2] Sharad Puri,[3] Menuka Adhikari,[1,4] Phadindra Wagle,[3] David N. McIlroy,[3] A. Kaan Kalkan,[2,*] and Yolanda Vasquez[1,*]

[1]Department of Chemistry, Oklahoma State University, Stillwater, OK 74078, United States

[2]Department of Mechanical and Aerospace Engineering, Oklahoma State University, Stillwater, OK 74078, United States

[3]Department of Physics, Oklahoma State University, Stillwater, OK 74078, United States

[4]Present Address: Department of Chemistry, Physics and Materials Sciences, Fayetteville State University, Fayetteville, NC 28301, United States

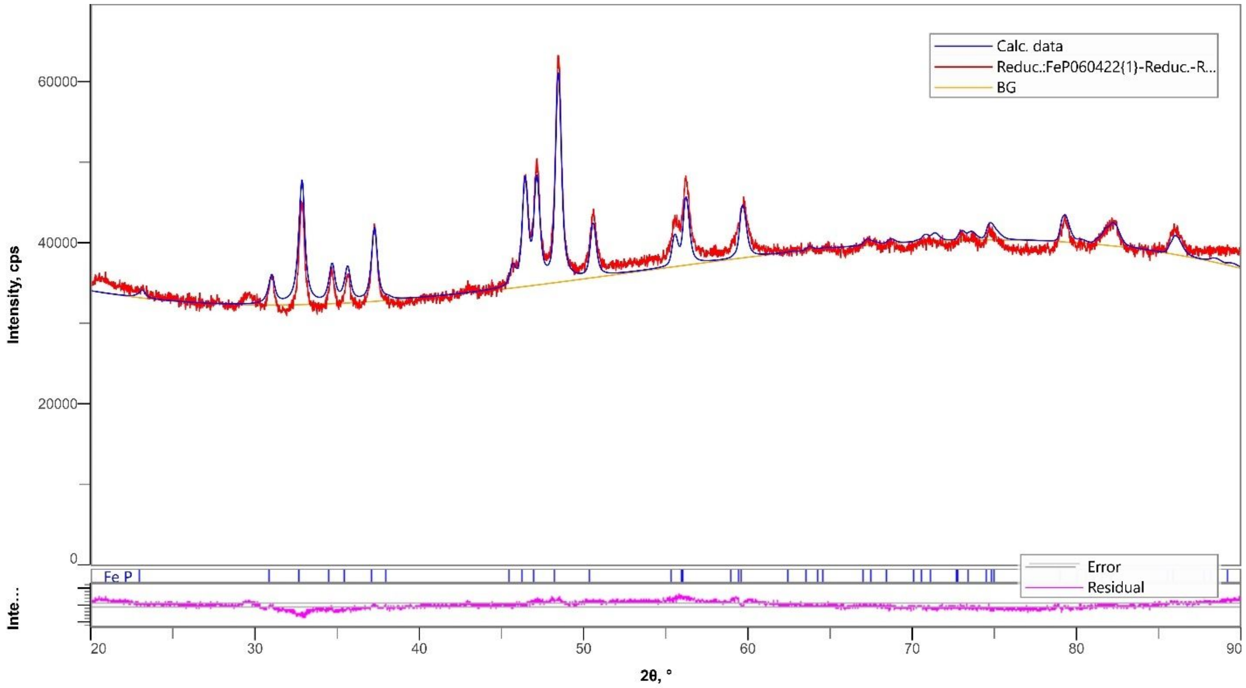

Figure S1. Whole powder pattern fitting profile of FeP nanobundles. The XRD pattern of the FeP nanobundles was fitted and refined by Rietveld refinement using Smart Lab Studio (Rigaku) software.

Table S1. Crystal data and structure refinement parameters for FeP nanobundles.

Data	Value
**Chemical Formula**	FeP
**Composition**	FeP
**Z-value**	1.00
**Concentration, wt%**	100.00
**RIR value**	2.348 (From structure)
**DB card number**	9016731
**Crystal system**	Orthorhombic
**Space group**	62: Pbnm
**a, Å**	5.76599
**b, Å**	5.17223
**c, Å**	3.09482
**α, °**	90.000
**β, °**	90.000
**γ, °**	90.000

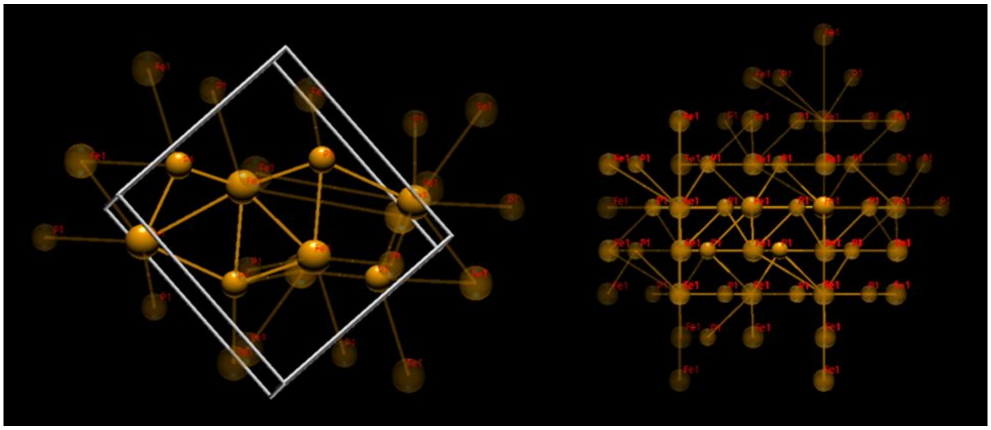

Figure S2. Side view of the FeP crystal structure along the b-axis and the corresponding view of the FeP packing structure along the c-axis.

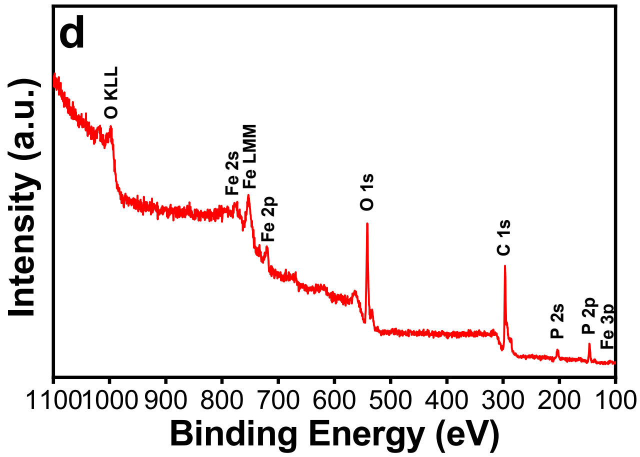

Figure S3. X-ray photoelectron spectroscopy survey spectrum of FeP nanobundles.

Table S2. BET surface area analysis profile.

Data	Value
**$SA_{(BET)}$**	4.123 sq. m/g
**Slope**	7.64669×10^2
**Intercept**	7.99355×10^1
**Corr**	0.9985
**C value**	1.057×10^1
**Pore Volume**	1.838×10^{-2} (cc/g)
**PSD**	Less than 2305.7 Å
**Pore Radius $_{(average)}$**	8.913×10^1 Å
**$SA_{(Langmuir)}$**	2.714×10^2 sq. m/g
**Slope**	1.283×10^1
**Intercept**	1.971×10^2
**Corr**	0.0721
**Micropore Volume**	0.000 cc/g
**Micropore Area**	0.000 sq. m/g
**Mesopore Area**	4.123 sq. m/g

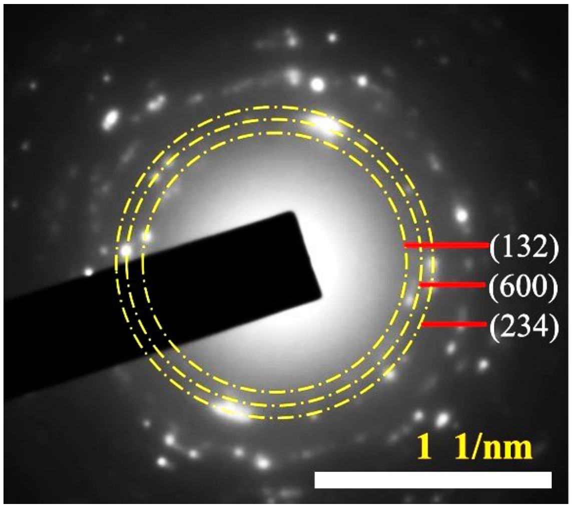

Figure S4. Selected-Area Electron Diffraction (SAED) pattern of as-synthesized FeP nanobundles. The pattern reveals discrete rings that can be indexed to the (132), (600), and (234) planes of the orthorhombic FeP nanobundles.

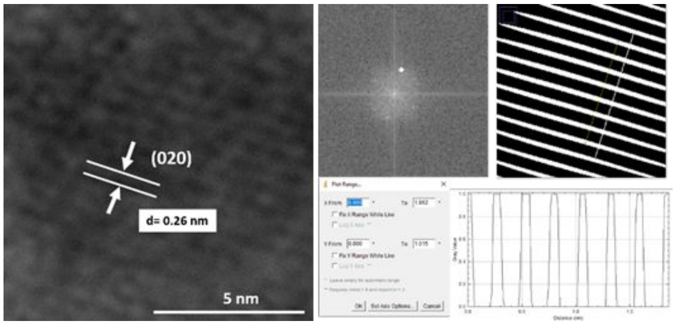

Figure S5. Lattice fringes indicate a lattice spacing of d_{002} = 0.29 nm, consistent with the orthorhombic phase of FeP.

Table S3. HER performance of transition metal phosphide (TMP) electrocatalysts in a 0.5 M H_2SO_4 electrolyte solution.

Catalyst	Morphology	Mass loading (mg/cm^2)	Current density (-j) (mA/cm^2)	Overpotential at corresponding current density	Tafel slope (mV/Dec)	Reference
**FeP**	Nanobundles	0.85	10	170	75	This work
**FeP/ NCNT**	Nanoparticles	0.36	10	113	59	[1]
**FeP_2/C**	Nanohybrid	0.425	5	500	66	[2]
**FeP**	Hollow microspheres	0.5	10	144	58	[3]
**FeP@C**	Hollow microcubes	0.72	10	115	56	[4]
**FeP/rGO**	Nanowires	0.204	10	107	58	[5]
**FeP**	Nanoparticles	0.36	10	292	86	[1]
**CoP/CNT**	Nanohybrid	0.285	10	122	54	[6]
**Ni_2P**	Hollow Nanoparticles	1	20	140	46	[7]
**FeP**	Nanosheets	0.285	10	240	67	[8]
**CoP**	Microspheres	0.285	10	226	76	[6]
**FeP NP@NPC**	Nanoparticles	1.45	10	130	67	[9]
**FeP**	Nanorods	0.565	10	107	54	[10]
**FeP**	Nanosheets	0.56	10	129	64	[10]
**FeP**	Super-structure	0.56	10	66	46	[10]
**MoP**	Nanoparticles	0.36	10	125	54	[11]
**CoP**	Nanoparticles	2	20	85	50	[12]
**CoP_x**	Nanoparticles	-	10	209	70	[13]
**Fe-Co_xP**	Nano-composite	-	10	127	55	[13]
**FeP**	Nanoparticles	-	10	172	68	[13]

Table S4. HER performance of transition metal electrocatalysts in KOH electrolyte solutions.

Catalyst	Mass loading (mg/cm^2)	Current density (-j) (mA/cm^2)	Overpotential at corresponding current density	Tafel slope (mV/Dec)	Reference
**FeP**	0.85	10	338	159	This Work
**FeP-CFP**	0.65	10	221	136	[14]
**Co_9S_8@NOSC**	0.28	10	320	105	[15]
**α-WP2**		-	259	165	[15]
**β-WP2**	-	-	277	180	[15]
**Co-NRCNTs**	0.28	10	370	-	[15]
**CoP@BCN**	-	10	215	52	[15]
**Pt-Sm**	-	100	366	169	[15]
**Pt-Ho**	-	100	414	131	[15]
**Pt-Ce**	-	100	390	114	[15]
**ONPPGC/OCC**	0.1	10	446	154	[15]

Table S5. Post-HER analysis of 0.5 M H_2SO_4 and 1 M KOH electrolytes (25 mL)

**Lab ID: 2189**	**ICAP-P ppm**	**Fe ppm**
0.5 M H2SO4 electrolyte	12.71	8.20
1 M KOH electrolyte	23.40	0.07

Note: Based on electrocatalyst (70% FeP) loading of 0.85 mg/cm^2, electrode area (1 cm^2), and electrolyte volume of 25 mL, we have computed percentages of Fe and P leached (based on the initial mass of Fe and P in the electrode) after 15 h of HER at 50 mA/cm^2. The percentage of the Fe and P leached are computed as: 0.054% and 0.00083% in 0.5 M H_2SO_4 and 0.083% and 0.28% in1 M KOH, respectively.

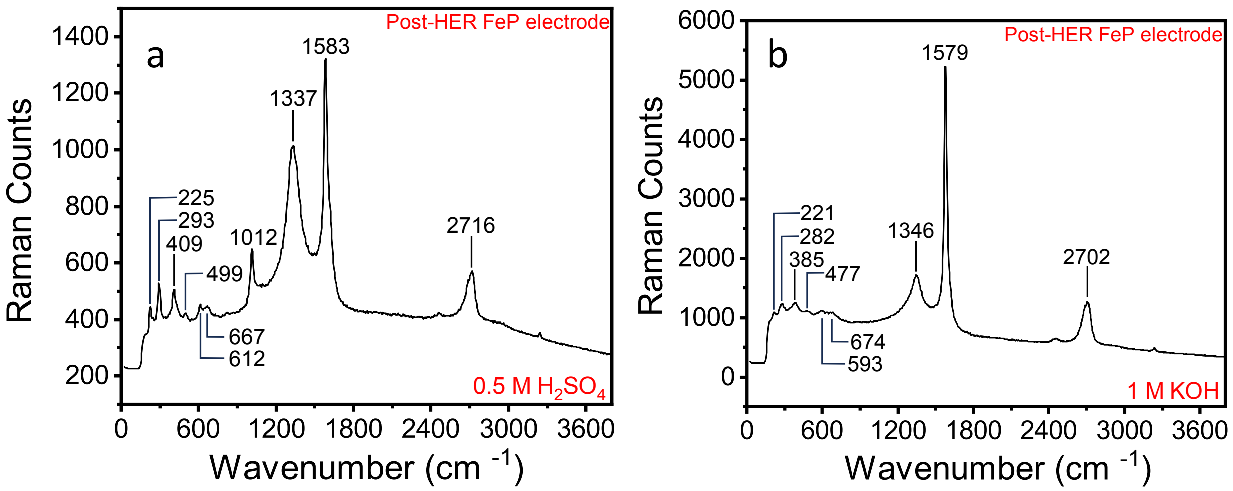

Figure S6. Post-HER Raman spectrum of FeP working electrode in 0.5 M H_2SO_4 (a) and 1 M KOH (b), respectively. Spectra were acquired with a 20× 0.4 NA lens and diffraction-limited laser beam spot size for 100 s. A laser power of 10 mW was employed. All other acquisition parameters are as in the main text.

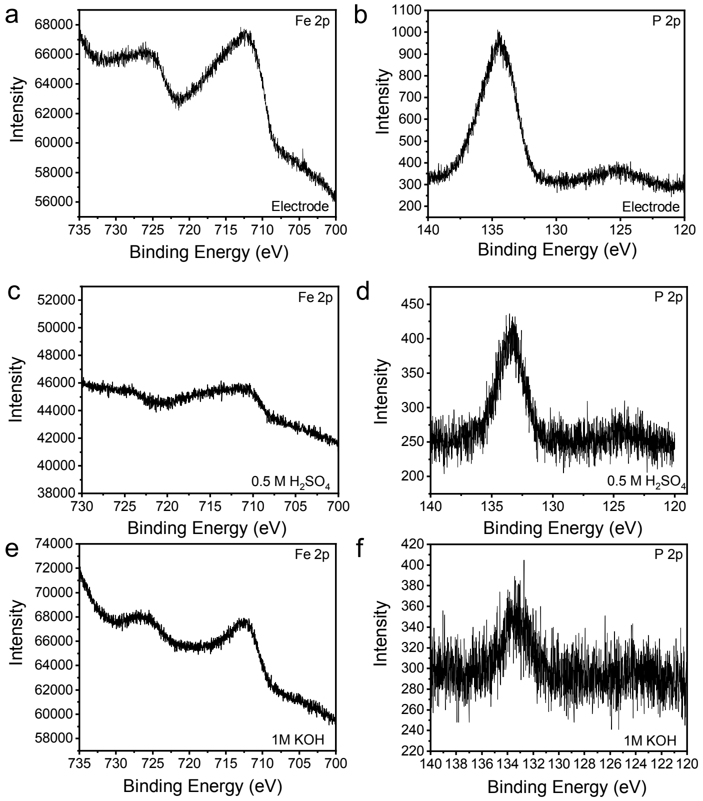

Figure S7. High-resolution XPS core-level spectra of FeP electrodes: (a) Fe 2p and (b) P 2p regions of the fresh electrode; (c) Fe 2p and (d) P 2p regions after 15 hours of testing in 0.5 M H_2SO_4; (e) Fe 2p and (f) P 2p regions after 15 hours of testing in 1 M KOH.

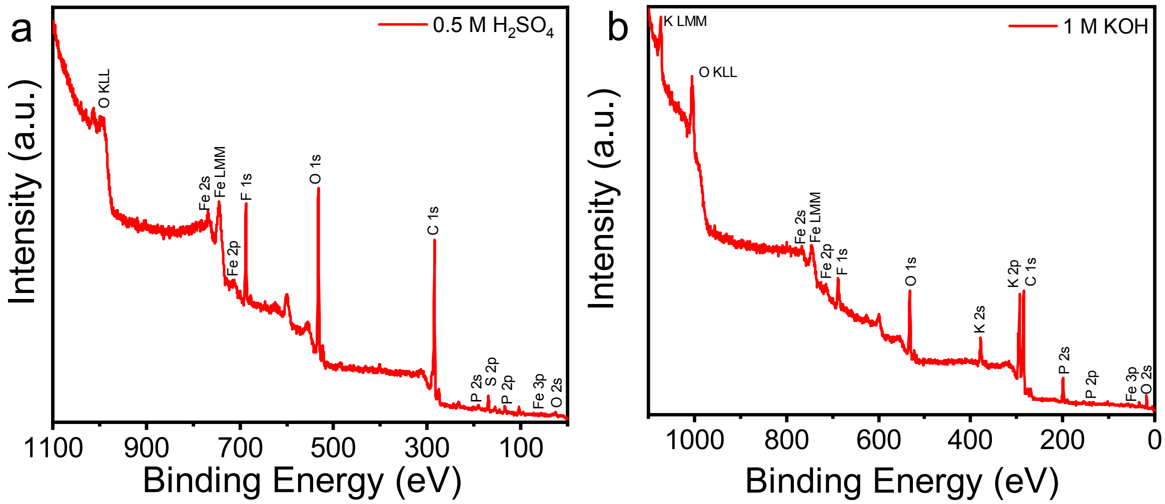

Figure S8. Post-HER XPS survey spectrum of FeP working electrode in 0.5 M H_2SO_4 (a), and 1 M KOH (b).

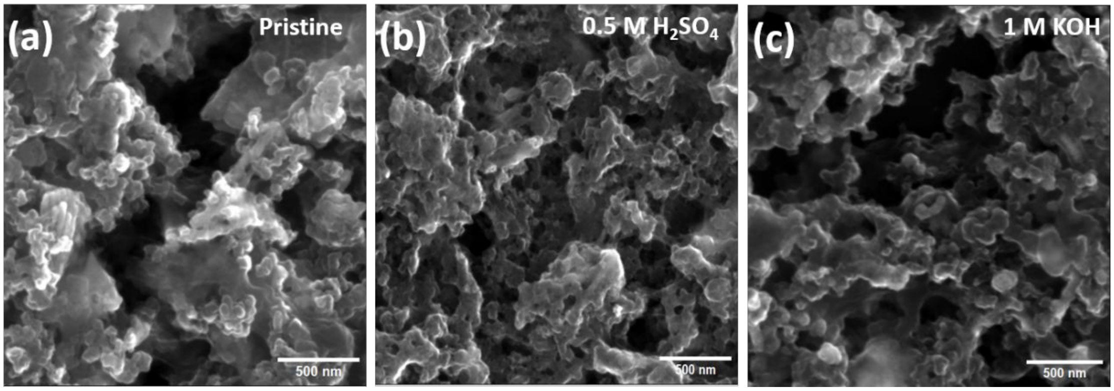

Figure S9. FE-SEM images of FeP electrodes: (a) before chronopotentiometry (pristine), and after chronopotentiometry in (b) 0.5 M H_2SO_4 and (b) 1M KOH.

Base(2)

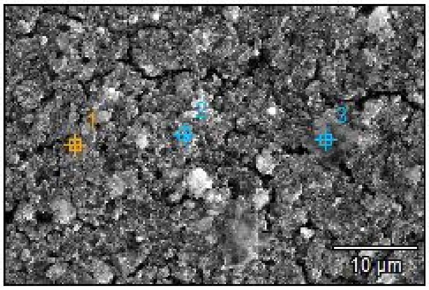

Image Name:	Base(2)
Image Resolution:	512 by 340
Image Pixel Size:	0.10 µm
Acc. Voltage:	20.0 kV
Magnification:	4079

Atomic %

	C	***O***	***Mg***	***Al***	***Si***	***P***	***Ca***	***Ti***	***Fe***	***Zn***
Base(2)_pt1	60.40	26.17			1.15	7.24	1.01		3.71	0.30
Base(2)_pt2	60.34	27.44	0.05		1.21	6.33	1.01		3.37	0.25
Base(2)_pt3	66.64	22.67		0.01	1.13	5.64	0.96	0.12	2.58	0.24

Figure S10. FE-SEM Energy Dispersive X-ray Spectroscopy data of a pristine FeP electrode (before chronopotentiometry).

Base(2)

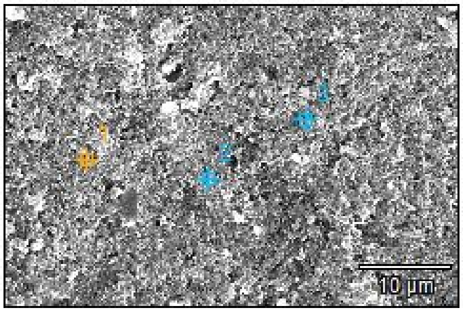

Image Name:	Base(2)
Image Resolution:	512 by 340
Image Pixel Size:	0.10 µm
Acc. Voltage:	20.0 kV
Magnification:	4079

Atomic %								
	C	***O***	***Al***	***Si***	***P***	***S***	***Ca***	***Fe***
Base(2)_pt1	91.58	6.21	0.04	0.59	0.74	0.32		0.53
Base(2)_pt2	84.86	12.12	0.05	1.32	0.73	0.40	0.04	0.48
Base(2)_pt3	79.81	16.22	0.04	1.21	1.39	0.22		1.11

Figure S11. FE-SEM Energy Dispersive X-ray Spectroscopy data of a FeP electrode after chronopotentiometry in H_2SO_4.

Base(2)

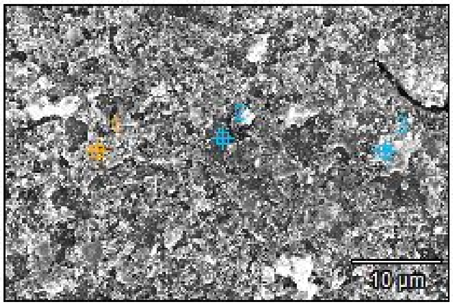

Image Name:	Base(2)
Image Resolution:	512 by 340
Image Pixel Size:	0.10 µm
Acc. Voltage:	20.0 kV
Magnification:	4079

Atomic %										
	C	***O***	***F***	***Mg***	***Si***	***P***	***S***	***Cl***	***K***	***Fe***
Base(2)_pt1	78.08	16.42			0.26	0.25	0.06	0.08	3.97	0.88
Base(2)_pt2	90.20	3.71	0.48		0.07	0.19		0.06	5.13	0.17
Base(2)_pt3	91.37	3.14		0.03	0.12	0.25		0.08	4.62	0.37

Figure S12. FE-SEM Energy Dispersive X-ray Spectroscopy data of a FeP electrode after chronopotentiometry in KOH.

References

1. Liu, Q.; Pu, Z.; Asiri, Abdullah, M.; Sun X. Nitrogen-doped carbon nanotube supported iron phosphide nanocomposites for highly active electrocatalysis of the hydrogen evolution reaction. *Electrochim. Acta.* **2014**, *149*, 324-329.
2. Jiang, J.; Wang, C.; Zhang, J.; Wang, W.; Zhou, X. Synthesis of FeP2/C nanohybrids and their performance for hydrogen evolution reaction. *J. Mater. Chem. A.* **2015**, *3*(2), 499-503.
3. Guo, X.; Feng, Z.; Lv, Z.; Bu, Y.; Liu, Q. Formation of Uniform FeP Hollow Microspheres Assembled by Nanosheets for Efficient Hydrogen Evolution Reaction. *ChemElectroChem.* **2017**, *4*(8), 2052-2058.
4. Zhu, X.; Liu, M.; Liu, Y.; Chen, R.; Nie, Z.; Li, J.; Yao, S. Carbon-coated hollow mesoporous FeP microcubes: an efficient and stable electrocatalyst for hydrogen evolution. *J. Mater. Chem. A.* **2016**, *4*(23), 8974-8977.
5. Yan, Y.; Thia, L.; Xia, Bao, Y.; Ge, X.; Liu, Z.; Fisher, A.; Wang, X. Construction of Efficient 3D Gas Evolution Electrocatalyst for Hydrogen Evolution: Porous FeP Nanowire Arrays on Graphene Sheets. *Adv. Sci.* **2015**, *2*(8), 1500120.
6. Liu, Q.; Tian, J.; Cui, W.; Jiang, P.; Cheng, N.; Asiri, Abdullah, M.; Sun, X. Carbon Nanotubes Decorated with CoP Nanocrystals: A Highly Active Non-Noble-Metal Nanohybrid Electrocatalyst for Hydrogen Evolution. *Angew. Chem. Int. Ed.* **2014**, *53*(26), 6710-6714.
7. Popczun, E.J.; McKone, J. R.; Read, C.G.; Biacchi, A. J.; Wiltrout, A. M.; lewis, N.S.; Schaak, R.E. Nanostructured Nickel Phosphide as an Electrocatalyst for the Hydrogen Evolution Reaction. *J. Am. Chem. Soc.* **2013**, *135*(25), 9267-9270.
8. Xu, Y.; Wu, R.; Zhang, J.; Shi, Y.; Zhang, B. Anion-exchange synthesis of nanoporous FeP nanosheets as electrocatalysts for hydrogen evolution reaction. *Chem. Comm.* **2013**, *49*(59), 6656-6658.
9. Pu, Z.; Amiinu, I. S.; Zhang, C.; Wang, M.; Kou, Z.; Mu, S. Phytic acid-derivative transition metal phosphides encapsulated in N,P-codoped carbon: an efficient and durable hydrogen evolution electrocatalyst in a wide pH range. Nanoscale, **2017**, *9*(10), 3555-3560.
10. Lin, C.; Gao, Z.; Yang, J.; Liu, B.; Jin, J. Porous superstructures constructed from ultrafine FeP nanoparticles for highly active and exceptionally stable hydrogen evolution reaction. *J. Mater. Chem. A.* **2018**, *6*(15), 6387-6392.
11. Xing, Z.; Liu, Q.; Asiri, Abdullah, M.; Sun, X. Closely Interconnected Network of Molybdenum Phosphide Nanoparticles: A Highly Efficient Electrocatalyst for Generating Hydrogen from Water. *Adv. Mater.* 2014, *26*(32), 5702-5707.
12. Popczun, E.J.; Read, C. G.; Roske, C. W.; Lewis, N. S.; Schaak, R. E. Highly Active Electrocatalysis of the Hydrogen Evolution Reaction by Cobalt Phosphide Nanoparticles. *Angew. Chem. Int. Ed.* **2014**, *53*(21), 5427-5430.
13. Guo, X.; Yu, X.; Feng, Z.; Liang, J.; Li, Q.; Lv, Z.; Liu, B.; Hao, C.; Li, G. Intercalation Synthesis of Prussian Blue Analogue Nanocone and Their Conversion into Fe-Doped CoxP Nanocone for Enhanced Hydrogen Evolution. *ACS Sustain. Chem. Eng.* **2018,** *6*(7), 8150-8158.
14. Lv, C.; Peng, Z.; Zhao, Y.; Huang, Z.; Zhang, C.; The hierarchical nanowires array of iron phosphide integrated on a carbon fiber paper as an effective electrocatalyst for hydrogen generation. *J. Mater. Chem. A.* **2016**, *4*(4), 1454-1460.
15. Mahmood, N.; Yao, Y.; Zhnag, J. W.; Pan, L.; Zhang, X.; Zou, J. J. Electrocatalysts for Hydrogen Evolution in Alkaline Electrolytes: Mechanisms, Challenges, and Prospective Solutions. *Adv. Sci.* **2018**. *5*(2), 1700464.
